# Heart Rate and Heart Rate Variability Assessment Identifies Individual Differences in Fear Response Magnitudes to Earthquake, Free Fall, and Air Puff in Mice

**DOI:** 10.1371/journal.pone.0093270

**Published:** 2014-03-25

**Authors:** Jun Liu, Wei Wei, Hui Kuang, Joe Z. Tsien, Fang Zhao

**Affiliations:** 1 Key Laboratory of Brain Functional Genomics (Ministry of Education), Institute of Brain Functional Genomics, East China Normal University, Shanghai, China; 2 Banna Biomedical Research Institute, Xi-Shuang-Ban-Na Prefecture, Yunnan, China; 3 Brain and Behavior Discovery Institute and Department of Neurology, Medical College of Georgia, Georgia Regents University, Augusta, Georgia, United States of America; Peking University, China

## Abstract

Fear behaviors and fear memories in rodents have been traditionally assessed by the amount of freezing upon the presentation of conditioned cues or unconditioned stimuli. However, many experiences, such as encountering earthquakes or accidental fall from tree branches, may produce long-lasting fear memories but are behaviorally difficult to measure using freezing parameters. Here, we have examined changes in heartbeat interval dynamics as physiological readout for assessing fearful reactions as mice were subjected to sudden air puff, free-fall drop inside a small elevator, and a laboratory-version earthquake. We showed that these fearful events rapidly increased heart rate (HR) with simultaneous reduction of heart rate variability (HRV). Cardiac changes can be further analyzed in details by measuring three distinct phases: namely, the rapid rising phase in HR, the maximum plateau phase during which HRV is greatly decreased, and the recovery phase during which HR gradually recovers to baseline values. We showed that durations of the maximum plateau phase and HR recovery speed were quite sensitive to habituation over repeated trials. Moreover, we have developed the fear resistance index based on specific cardiac response features. We demonstrated that the fear resistance index remained largely consistent across distinct fearful events in a given animal, thereby enabling us to compare and rank individual mouse’s fear responsiveness among the group. Therefore, the fear resistance index described here can represent a useful parameter for measuring personality traits or individual differences in stress-susceptibility in both wild-type mice and post-traumatic stress disorder (PTSD) models.

## Introduction

Emotionally charged episodic events can often produce robust and long-lasting memories even upon a single exposure [1]–[3]. Extensive behavioral, lesion and brain imaging studies have shown that emotion can facilitate the encoding, consolidation and retrieval of memory [4]–[7]. In addition, emotional stimuli, especially the fearful/aversive stimuli, typically evoke innate unconditioned responses [8]–[11]. Therefore, many fearful stimuli have been used as unconditioned stimuli in classical fear conditioning to study the neural mechanism of learning and memory [12]–[17] and are useful probes for investigation of emotions [18], [19].

In rodents, freezing behavior has been widely used to assess emotional responses elicited by fearful stimuli. Freezing is defined as the absence of body movement except for breathing in rodents [9], [10]. The other type of response, startle response, which consists of a contraction of the skeletal, facial and neck muscles, is also used to assess fear [20]–[23]. In humans, the eye blink behavior as a startle response to air puff to eyes is typically measured by electromyography (EMG) of the orbicularis oculi muscle [24]. In rats and mice, stabilimeter and accelerometer devices are also used to record the amplitude of the startle response to fearful events [25]–[27].

Recently, we have used our large-scale neural ensemble recording technique to monitor the real-time encoding patterns of fearful experiences in hippocampal CA1 region and anterior cingulate cortex (ACC) of freely behaving mice [28]–[30]. We have shown that different fearful events, such as air puff, free fall, and earthquake-like shake, evoked diverse responses in CA1 and ACC neurons, consequently permitting quantitative measurement of the formation of real-time neural ensemble traces of these fearful experiences in the mouse brain [28]–[30]. In addition, we have shown that earthquake and free fall can evoke robust suppression-and-offset-rebound excitation of the ventral tegmental area (VTA) putative dopamine neurons in freely behaving mice [31], indicating the termination of aversive or fearful events are strong drive for increasinging dopamine activity in the brain. Unfortunately, the traditional behavioral responses, such as the freezing behavior, can’t be used to quantitatively measure fearfulness evoked by earthquake, free fall, or air puff because animals often move and exhibit various escape behaviors during or immediately after these events. Similarly, the transient startle responses [2], [26], measured by stabilimeter and accelerometer devices, are not suitable to provide continuous assessments under such experimental paradigms.

These limitations have prompted us to examine autonomic responses, such as changes of heartbeat, as a method reported in the literature to assess behavioral changes in rats [32]–[36]. HR and HRV can be determined from electrocardiogram (ECG) generated by the sinoatrial node. It has been shown that changes of HR and HRV are correlated with emotional changes in animals, such as fear in both mice and rats [37]–[39]. In addition, several early works in rats also reported HR changes to startling acoustic sound or air puff stimuli [33]–[35], [40], [41]. Consistent with these findings, our recent mouse study has further demonstrated that fear conditioning can trigger strong changes in HR and HRV in freely behaving mice [42]. In particular, we have further shown that HRV is highly correlated with the performances in both 1-h short-term and 24-h long-term fear memory tests [42].

In the present study, we set out to examine whether and how emotionally fearful events, such as earthquake, free fall, and air puff, would trigger cardiac changes in freely behaving mice. We have systematically and quantitatively characterized the various stages of HR responses to air puff, free fall, and earthquake-like cage-shake in freely behaving mice. Moreover, we have examined whether and how multiple trials or exposures to such fearful events produce habituation-like changes in HR and HRV dynamics. Finally, we have employed the fear resistance index to describe and compare the summed autonomic responses across individual mice.

## Results

### Three Distinct Heart Rate Response Phases to Fearful Stimuli in Freely Behaving Mice

**Figure 1:**
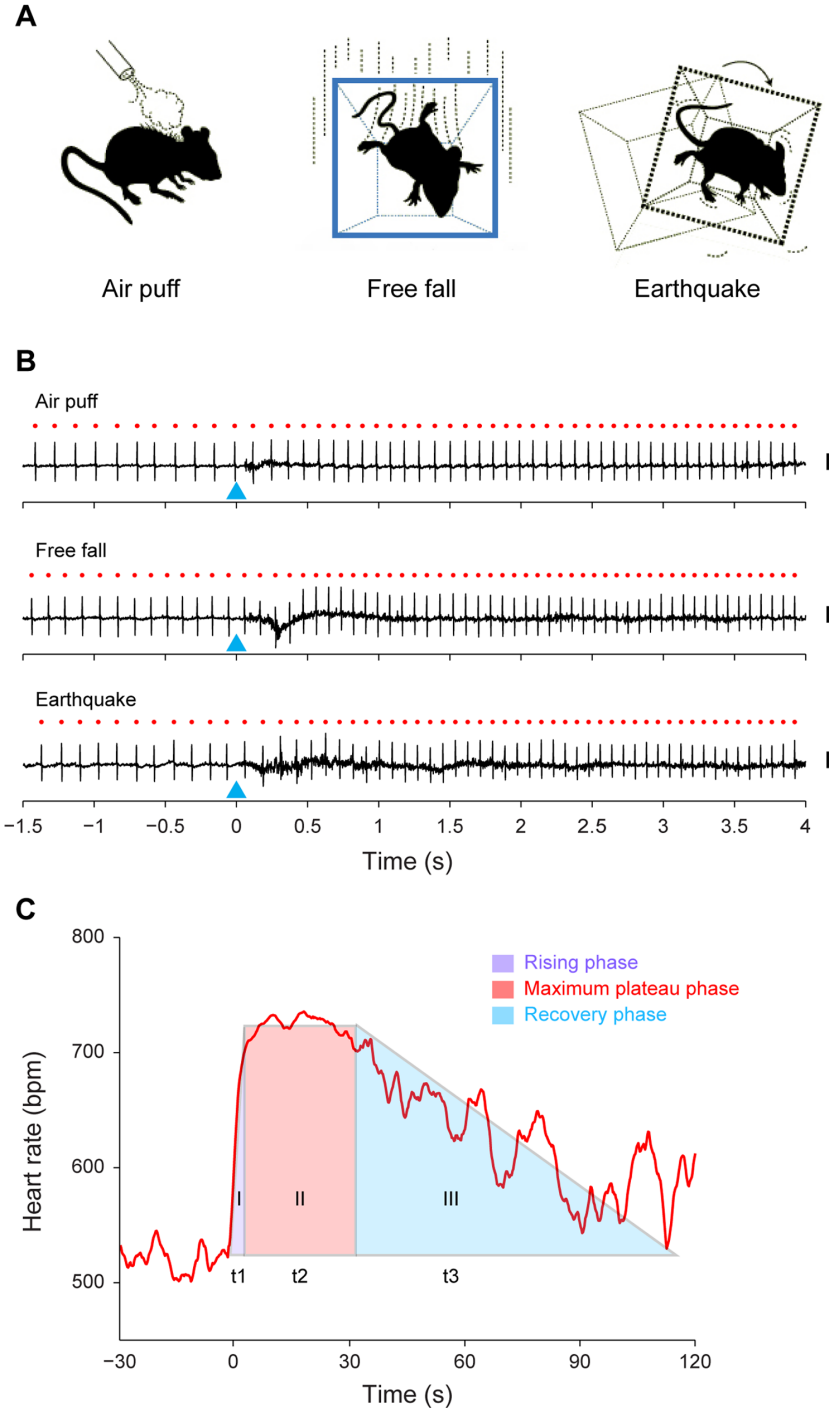
ECG recording and three distinct phases of HR responses to fearful stimuli. (A) Illustration of three fearful stimuli paradigms: namely, air puff, a sudden air blow (10 psi, 0.5 s) to the back of the animal; free fall, an abrupt drop of the animal inside a small chamber (13 cm high); and earthquake, an unexpected earthquake-like shaking to the animal (3000 rpm, 0.5 s). (B) Examples of ECG recording during air puff, free fall and earthquake stimuli. The red dots indicate the peaks of the R-wave. The blue triangles indicate the onsets of each stimulus. Please note the significant shortened R-R intervals after the onset of all the three types of stimuli. Although noise was introduced during the presence of the stimuli, the custom-written Matlab program can still precisely extract the timestamps of the R-wave peaks. Scales: 0.5 mV. (C) Schematic representation of the three distinct phases of HR responses to fearful stimuli: first (t1, the purple triangle), the rising phase; second (t2, the red rectangle), the maximum plateau phase; and third (t3, the cyan triangle), the recovery phase.

To investigate whether and how air puff, free fall, and earthquake alter the emotional states, we set out to measure the changes in HR and HRV in freely behaving mice. We implanted a pair of electrodes subcutaneously into the chest of these mice for ECG recording using the procedures previously described [42]. Data from seven mice, in which the ECG signals were stably recorded, were used for the current analyses. The ECG data showed that air puff, free fall, and earthquake stimuli all triggered significant increases in HR (Figure 1A & B).

Moreover, we found that there were three distinct phases of HR responses to these stimuli: the rising phase, the maximum plateau phase and the recovery phase (Figure 1C). The rising phase was between the onset of stimulus and the start of HR plateau; the plateau phase was the stable period of maximum HR during which HRV was greatly reduced; and the recovery phase was the duration for HR gradually coming back to the basal level (see methods for details).

### Changes in Heart Rate and Heart Rate Variability in Response to Sudden Air Puff

**Figure 2:**
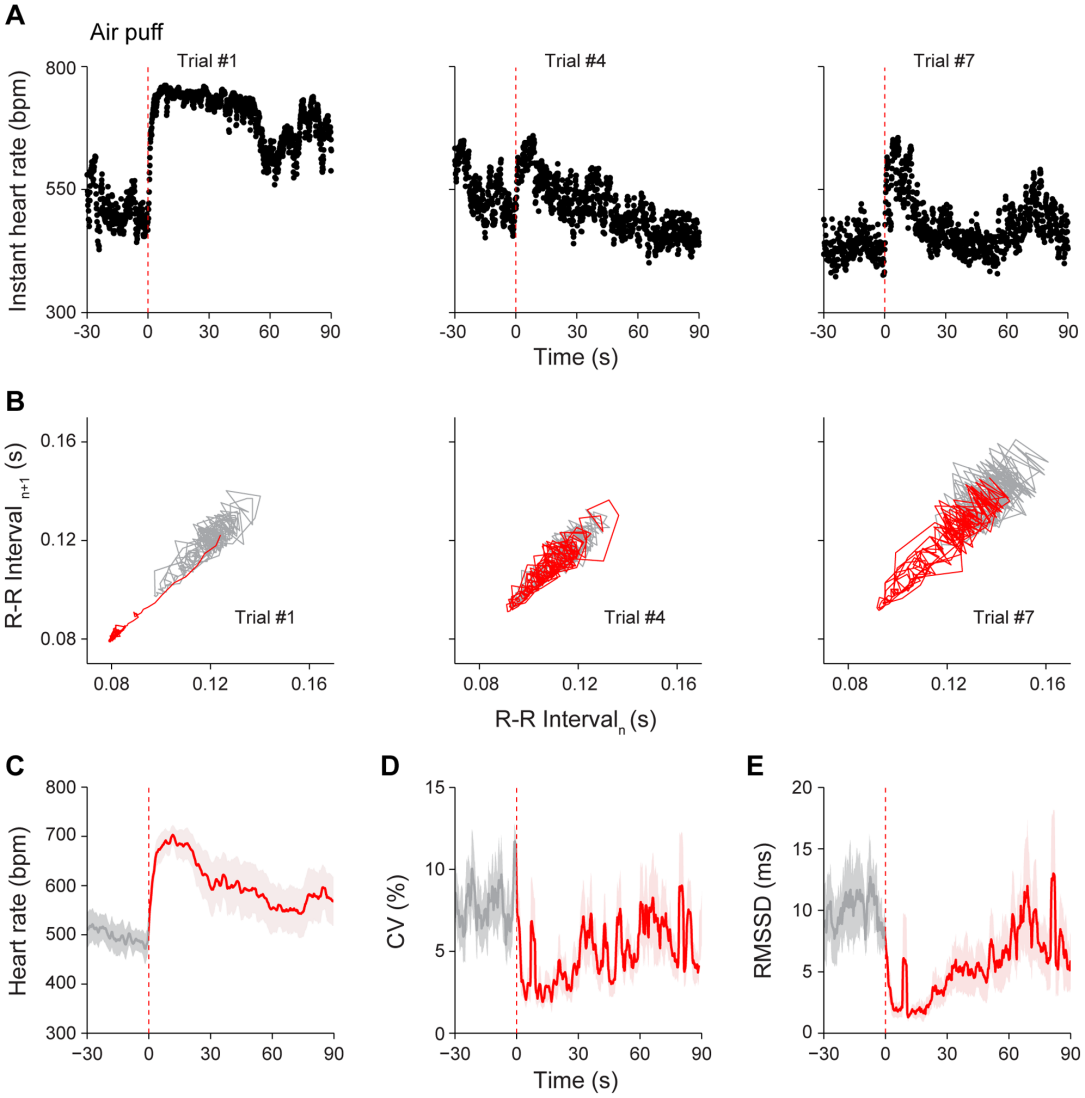
Response of HR dynamics to air puff. (A) Instant HR responses of an individual mouse during three trials of air puff stimuli, trial #1, trial #4 and trial #7. The red vertical dashed lines indicate the onset of the air puff stimuli (10 psi, 0.5 s). Robust increase of instant HR after stimulus onset was noted at trial #1. (B) Poincaré plots of the same mouse’s R-R intervals of 30-s pre-stimulus (grey line) and 30-s post-stimulus (red line) period in trial #1, trial #4 and trial #7. Prominent increase in heartbeat regularity during the 30-s post-stimulus period was noted in trial #1. (C) The average HR response to air puff (1^st^ trial). (D) The average CV of R-R intervals upon 1^st^ trial of air puff. (E) The average RMSSD of R-R intervals upon 1^st^ trial of air puff. The average curves of 30-s pre-stimulus period were plotted as grey lines, whereas the average curves of 90-s post-stimulus period were plotted as red lines. The shadows around the average curves indicate error bars (SEM); n = 7.

To study how the dynamics of HR and HRV would respond to air puff, we delivered a sudden air blow (10 psi for 0.5 s) to the back of the mouse, and repeated for seven times with 2–5 min randomized intervals between each air puff. In previous startle (air puff) studies in rats, both the tachycardia and bradycardia have been reported [33], [40], [41]. However, we only observed the tachycardia, a robust increase of instant HR after the onset of air puff stimulus in mice (Figure 2A, 1^st^ Trial). It is noted that repeated air puff to the animals reduced its effectiveness in eliciting cardiac changes, indicating the habituation effect (Figure 2A, 4^th^ and 7^th^ Trials). In order to quantitatively measure the real-time HRV, we performed a nonlinear dynamic technique, Poincaré plot analysis, which can be applied for visualization of the R-R interval fluctuation and assessment of the dynamics of HRV. Poincaré plot of R-R intervals revealed prominent increase in heartbeat regularity (or reduction in HRV) during the 30-s post-stimulus period (Figure 2B, the left panel indicates the response to 1^st^ Trial). The Poincaré plot analysis revealed the habituation effect over seven trials (Figure 2B, the middle and right panels showed cardiac responses to the 4^th^ and 7^th^ Trials, respectively). The mean HR responses from the seven mice showed that HR increased significantly from the basal level of 494±27 bpm to 690±24 bpm to the first air puff (*P*<0.001) (Figure 2C). Moreover, analysis of the averaged coefficient of variation (CV) of R-R intervals, measurements of HRV, has revealed the noticeable decrease after the 0.5-s air puff (Figure 2D). In addition, the root mean square of the successive differences (RMSSD) of R-R time intervals also confirmed decrease in HRV (Figure 2E).

**Figure 3:**
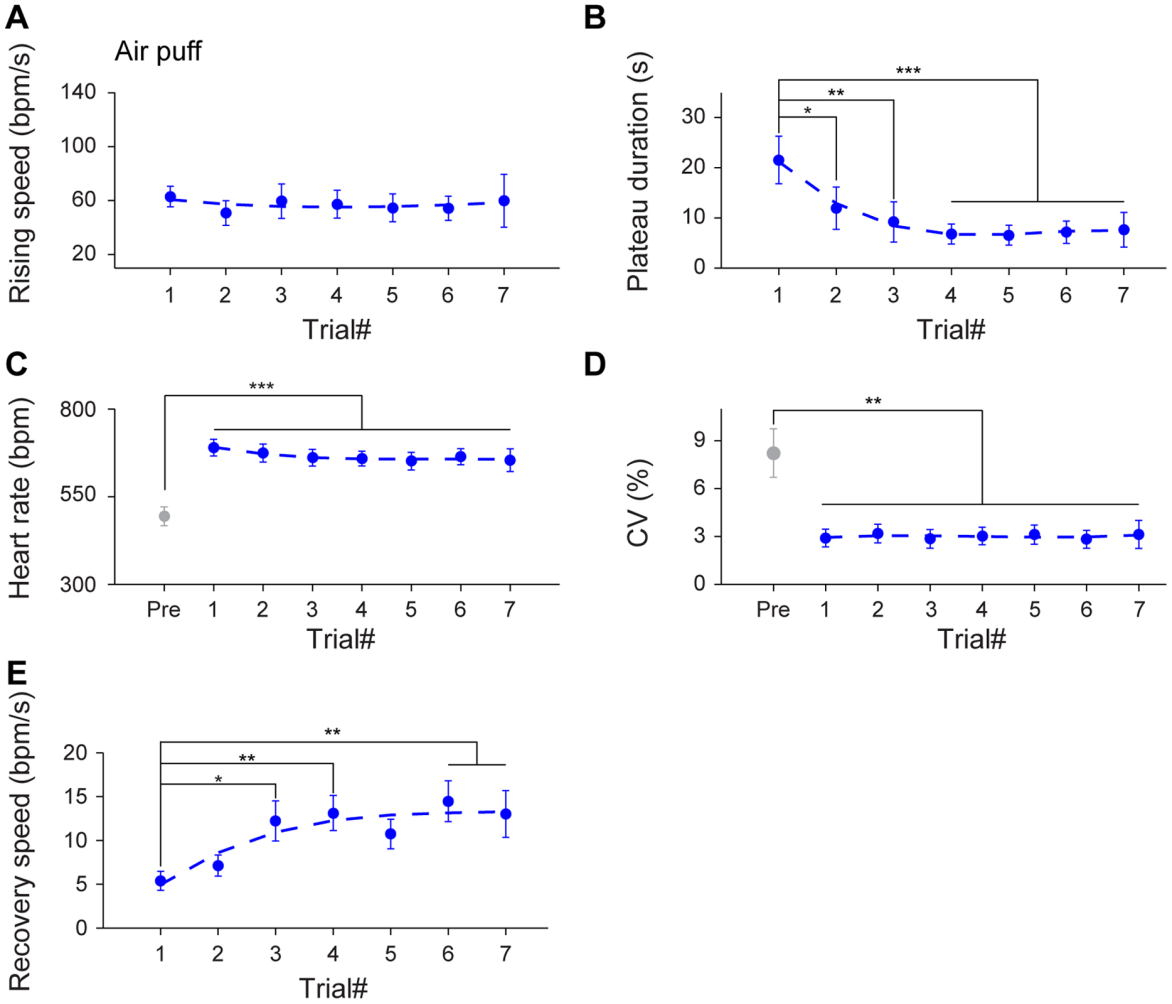
Effects of habituation on HR dynamics upon multiple trials of air puff. (A) The average rising speed showed no significant difference during seven trials of air puff stimuli. n = 7. (B) The plateau durations of seven air puff stimuli exhibited a significant decreasing trend from the second trial and there afterwards. n = 7; *P<0.05, **P<0.01, ***P<0.001, one-way repeated measures ANOVA and Dunnett’s multiple comparisons test. (C–D) In comparison to the pre-stimulus state, the significant changes of HR and CV during the maximum plateau maintained throughout seven trials. n = 7; **P<0.01, ***P<0.001, one-way repeated measures ANOVA, Tukey *post hoc* test and Dunnett’s multiple comparisons test. (E) The recovery speed across seven air puff stimuli showed a significant increase as air puff was repeated. n = 7; *P<0.05, **P<0.01, one-way repeated measures ANOVA and Dunnett’s multiple comparisons test. All data are plotted as mean ± SEM (error bars).

To provide further quantitative measurement of the habituation effects on the dynamic HR responses to air puff, we evaluated five distinct components of cardiac responses: namely, (1) the rising speed (of the rising phase) which characterizes the speed of the elevation of HR upon stimulation; (2) time duration of the maximum plateau; (3) HR of the maximum plateau; (4) CV of the maximum plateau; and (5) recovery speed (of the recovery phase). Interestingly, our analysis suggests that the rising speed did not change significantly throughout seven trials (Figure 3A, *F*_6, 36_ = 0.14, *P*>0.05). The maximum plateau duration time showed a significant decrease from the second trial and there afterwards in comparison to that of the first trial (Figure 3B, *F*_6, 36_ = 6.55, *P*<0.05, *P*<0.01, *P*<0.001). Yet, HR was consistently increased over the baseline after receiving air puff across all seven trials (Figure 3C, *F*_7, 42_ = 14.08, *P*<0.001), while CV in the maximum plateau phase exhibited dramatic and consistent decrease over the baseline after receiving air puff (Figure 3D, *F*_7, 42_ = 5.50, *P*<0.01). By contrast, the recovery speed showed a statistically significant increasing trend as air puff was repeated (Figure 3E, *F*_6, 36_ = 4.71, *P*<0.05, *P*<0.01). These observations showed that distinct cardiac parameters exhibited different responses to repeated air puff stimulation. In particular, the time duration of the maximum plateau phase and the HR recovery speed were quite sensitive for detecting habituation effects over multiple trials.

### Changes in Heart Rate and Heart Rate Variability in Response to Free Fall

**Figure 4:**
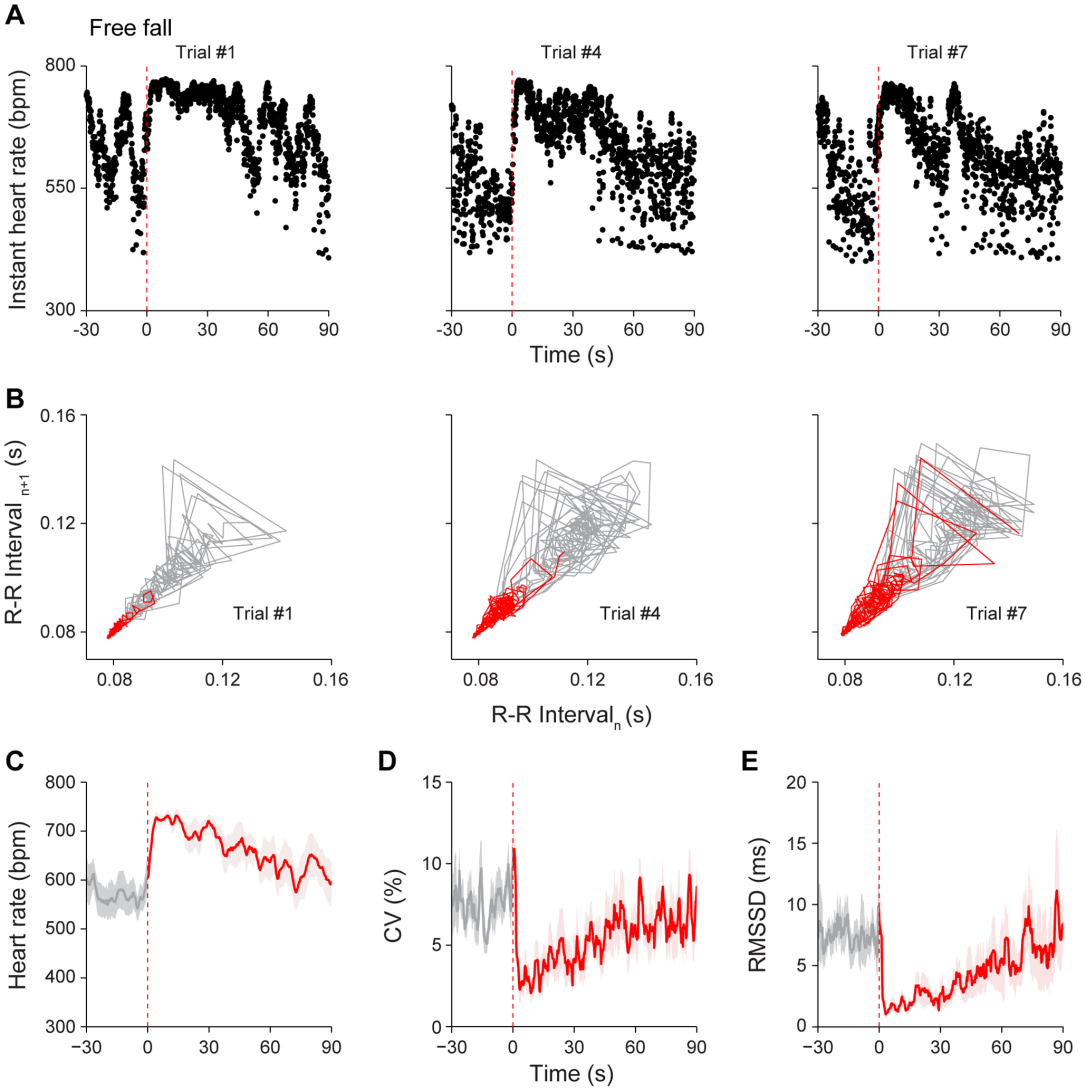
Response of HR dynamics to free fall. (A) Instant HR responses of an individual mouse during three free fall stimuli, trial #1, trial #4 and trial #7. The red vertical dashed lines indicate the onset of the free fall stimuli (13 cm high). (B) Poincaré plots of the same mouse’s R-R intervals of 30-s pre-stimulus (grey line) and 30-s post-stimulus (red line) period in trial #1, trial #4 and trial #7. Immediate decrease in dynamic patterns in HRV after stimulus onset was noted at trial #1, but more diffused distribution in the 30-s post-stimulus period in trial #4 and trial #7. (C) The average HR response to free fall (1^st^ trial). (D) The average CV of R-R intervals upon 1^st^ trial of free fall. (E) The average RMSSD of R-R intervals upon 1^st^ trial of free fall. The average curves of 30-s pre-stimulus period were plotted as grey lines, whereas the average curves of 90-s post-stimulus period were plotted as red lines. The shadows around the average curves indicate error bars (SEM); n = 7.

A sudden free fall can be an intense fearful event which mimics the tower-of-terror experience in Disney world for humans or the accidental fall from a tree branch for small animals. Our recent *in vivo* neural recording in mice revealed that free fall triggered dynamic firing changes in many neurons in the CA1 region of the mouse hippocampus [28] and also triggered firing changes in three types of the VTA dopaminergic neurons [31]. However, changes in HR dynamics during free fall in rodents have not been investigated. Thus, to measure changes in cardiac responses to free fall (or elevator drop), we placed the mouse in a small chamber which was 13 cm off the ground. In order to minimize the effect of novel environment on basal HR [42], mice were habituated to this chamber for three days (1 h each day). On the day of the experiments, we delivered a computerized signal to initiate the drop, and both HR and HRV were measured. The trials were repeated for seven times with 2–5 min randomized time intervals between each drop trial. We found that the instant HR increased in response to drop episodes (Figure 4A). Typically, the cardiac responses became smaller over repeated trials (Figure 4A). The Poincaré plot analysis also showed dynamic patterns in HRV decrease immediately and remained so for a certain period of time (Figure 4B). As shown in the Figure 4B, the initial 30 s (in red) in the first trial showed the tight R-R time interval distribution in this representative mouse (the left panel). However, the same 30-s periods from the 4^th^ and 7^th^ drop showed more diffused distribution (the middle and right panels, respectively). As a group, HR showed significant increases upon free fall drop. For example, the averaged HR was up-regulated from 567±28 bpm at the basal level to 731±9 bpm, (*P*<0.001) upon the 1^st^ drop, and then gradually decreased over the next 90 s (Figure 4C). For the same period of time, the averaged coefficient of variation (CV) of R-R intervals showed a decrease upon the drop (Figure 4D). Similarly, the root mean square of the successive differences of R-R time intervals also showed a decrease in HRV (Figure 4E).

**Figure 5:**
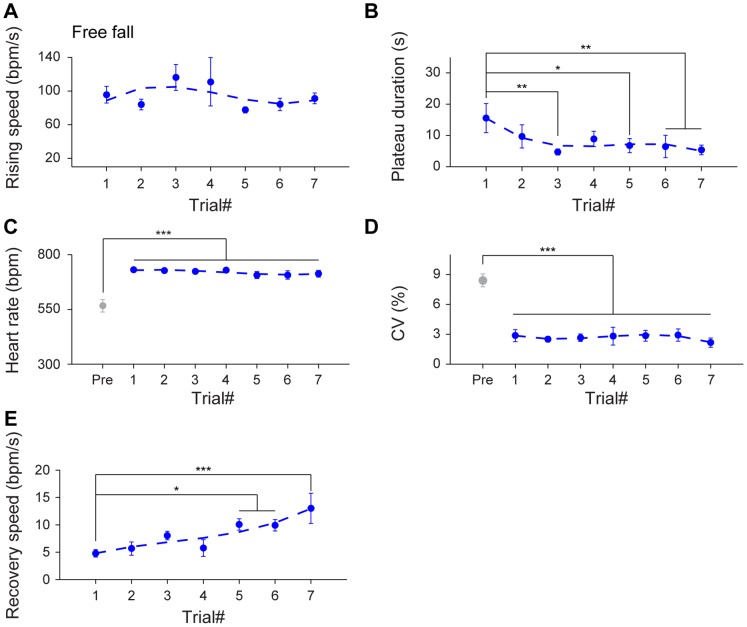
Effects of habituation on HR dynamics upon multiple trials of free fall. (A) The average rising speed showed no significant difference during seven trials of free fall. n = 7. (B) The time duration of the maximum plateau displayed a significant decrease over trials. n = 7; *P<0.05, **P<0.01, one-way repeated measures ANOVA and Dunnett’s multiple comparisons test. (C–D) In comparison to the pre-stimulus state, the significant changes of HR and CV during the maximum plateau maintained throughout seven trials. n = 7; ***P<0.001, one-way repeated measures ANOVA, Tukey *post hoc* test and Dunnett’s multiple comparisons test. (E) The recovery speed across seven free fall stimuli showed a significant tendency in becoming faster by the 5^th^ trial and afterwards. n = 7; *P<0.05, ***P<0.001, one-way repeated measures ANOVA and Dunnett’s multiple comparisons test. All data are plotted as mean ± SEM (error bars).

To examine in details the effects of multiple free fall events on HR changes, we evaluated the rising speed, time durations of the maximum plateau, HR and CV of the maximum plateau, and recovery speed over multiple drop trials. Although there was no habituation trend in HR acceleration (Figure 5A, *F*_6, 36_ = 1.19, *P*>0.05), the time durations of the maximum plateau phase showed significant decreases over seven trials (Figure 5B, *F*_6, 36_ = 3.72, *P*<0.05, *P*<0.01, *P*<0.001). The elevation of HR and reduction of CV continued throughout seven trials (Figure 5C & D, *F*_7, 42_ = 24.60, *P*<0.001, *F*_7, 42_ = 15.44, *P*<0.001). In addition, as a group, the recovery speed of the HR back to the basal level also significantly increased by the 5^th^ trial and afterwards (Figure 5E, *F*_6, 36_ = 5.43, *P*<0.05, *P*<0.001). Therefore, these detailed analyses revealed that while the HR and HRV continually showed significant differences over the basal levels, the time durations of the maximum plateau and the recovery speed were changing as drop was repeated over seven trials.

### Changes in Heart Rate and Heart Rate Variability in Response to Earthquake

**Figure 6:**
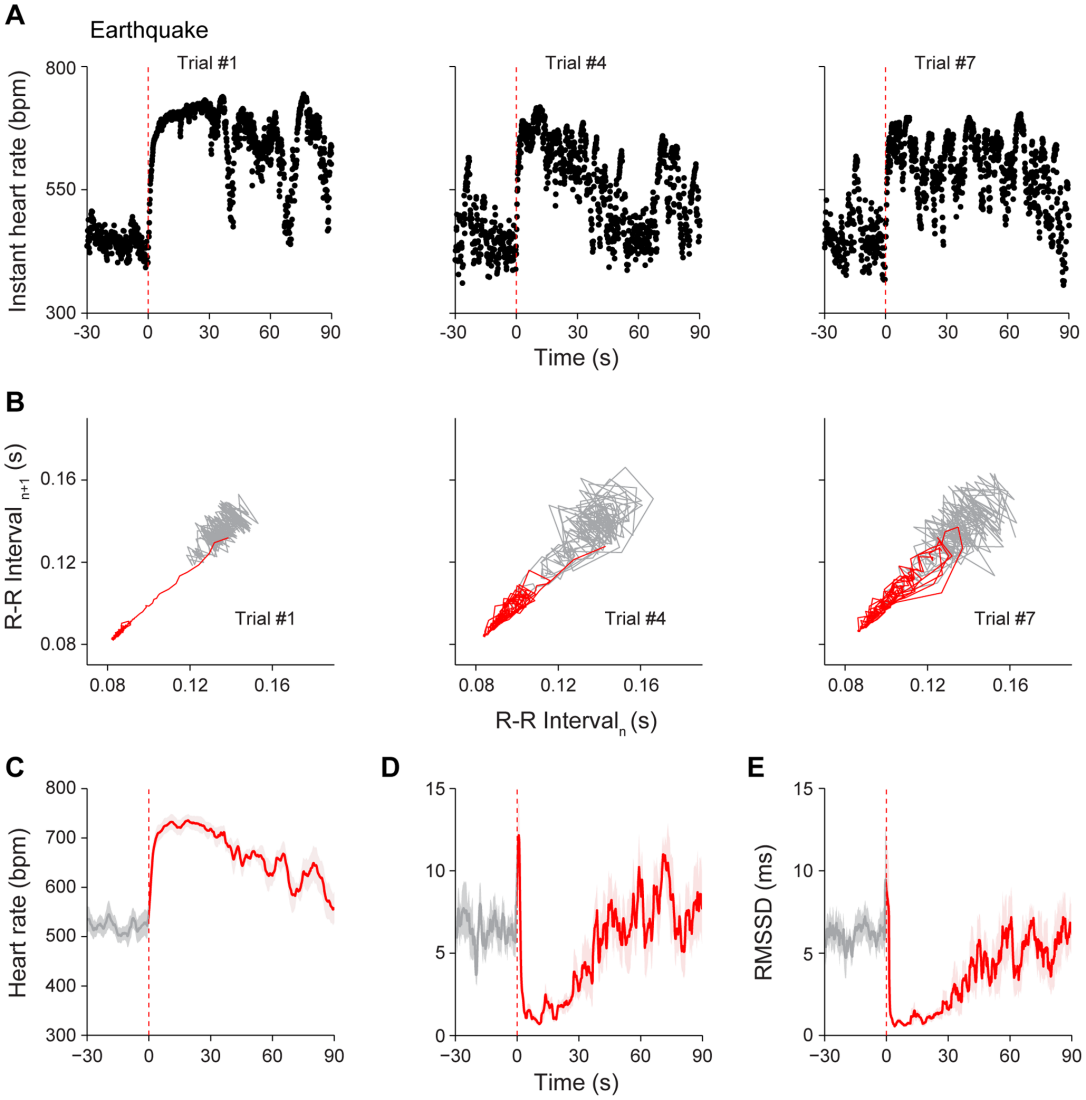
Response of HR dynamics to earthquake. (A) Instant HR responses of an individual mouse during three trials of earthquake stimuli, trial #1, trial #4 and trial #7. The red vertical dashed lines indicate the onset of the earthquake (0.5 s). (B) Poincaré plots of the same mouse’s R-R intervals of 30-s pre-stimulus (grey line) and 30-s post-stimulus (red line) period in trial #1, trial #4 and trial #7. Note the reduction of HRV in response to earthquake at trial #1, and such reduction effect became weaker at trial #4 and trial #7. (C) The average HR response to earthquake (1^st^ trial). (D) The average CV of R-R intervals upon 1^st^ trial of earthquake. (E) The average RMSSD of R-R intervals upon 1^st^ trial of earthquake. The average curves of 30-s pre-stimulus period were plotted as grey lines, whereas the average curves of 90-s post-stimulus period were plotted as red lines. The shadows around the average curves indicate error bars (SEM); n = 7.

To examine how earthquake events alter the dynamic HR responses, we placed mice in a small circular chamber which was fixed on top of a vortex machine, and earthquake event was triggered by a computerized program. Again, the mice underwent 1-hr habituation session each day for three days to minimize effects of the novel chamber. On the day of experiments, the mice were subjected to seven trials of earthquakes with 2–5 min of randomized time intervals between each trial. The instant HR showed the rapid tachycardia to earthquake. Responses of a representative mouse to the 1^st^, 4^th^ and 7^th^ shake trials are shown in Figure 6A (from the left to right panels). Although the earthquake occurred for only 500 ms, the duration of the maximum plateau phase could last up to 30 s (i.e. the first earthquake trial). The reduction in HRV in response to earthquake was further evident from Poincaré plot analysis of the given individual mouse (Figure 6B, the initial 30-s R-R time distribution plot from the 1^st^ Trial, red line). On average, HR increased from basal level of 518±18 bpm to 725±15 bpm upon the 1^st^ earthquake (*P*<0.001) (Figure 6C). Moreover, the averaged CV and RMSSD of R-R intervals also showed reductions in HRV (Figure 6D & E).

**Figure 7:**
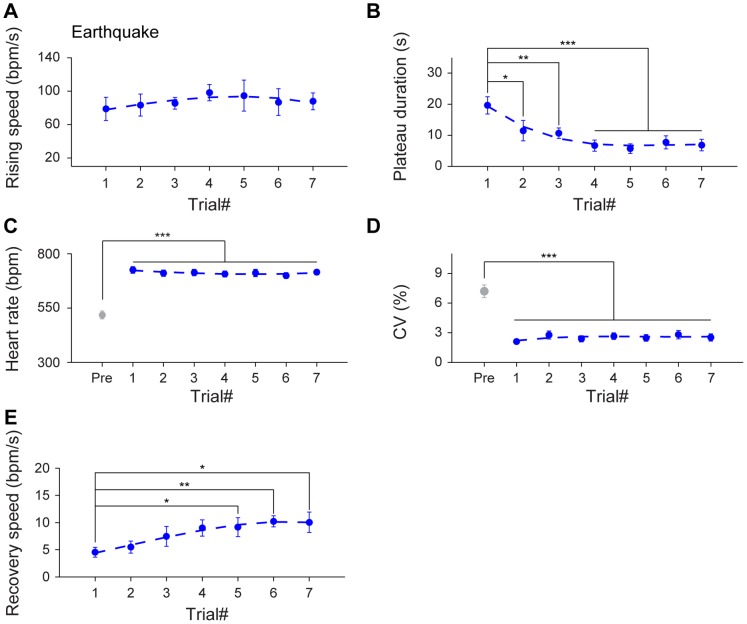
Effects of habituation on HR dynamics upon multiple trials of earthquake. (A) The average rising speed showed no significant difference during seven trials of earthquake stimuli. n = 7. (B) The plateau durations of seven earthquake stimuli decreased significantly from the second trial and afterwards. n = 7; *P<0.05, **P<0.01, ***P<0.001, one-way repeated measures ANOVA and Dunnett’s multiple comparisons test. (C–D) In comparison to the pre-stimulus state, the significant changes of HR and CV during the maximum plateau maintained throughout seven trials. n = 7; ***P<0.001, one-way repeated measures ANOVA, Tukey *post hoc* test and Dunnett’s multiple comparisons test. (E) The recovery speed across seven earthquake stimuli exhibited a significant increasing trend over trials. n = 7; *P<0.05, **P<0.01, one-way repeated measures ANOVA and Dunnett’s multiple comparisons test. All data are plotted as mean ± SEM (error bars).

Furthermore, we have systematically examined the various parameters on dynamic HR responses upon multiple trials of earthquake. The slope of HR increase maintained similar across trials (Figure 7A, *F*_6, 36_ = 0.79, *P*>0.05). However, time durations for the maximum plateau phase decreased significantly from the second earthquake trial and then afterwards (Figure 7B, *F*_6, 36_ = 7.71, *P*<0.05, *P*<0.01, *P*<0.001). Interestingly, the HR and CV of maximum plateau still existed significant responses throughout seven trials (Figure 7C & D, *F*_7, 42_ = 66.70, *P*<0.001, *F*_7, 42_ = 20.46, *P*<0.001). On the other hand, the recovery speed exhibited a tendency in becoming quicker over trials, with statistically differences at the 5^th^ trial and afterwards (Figure 7E, *F*_6, 36_ = 3.59, *P*<0.05, *P*<0.01.).

### Measurement of Summed Fear Responses to Different Events among Individual Animals

Under many circumstances, it may be highly desirable to compare the fear responses among different animals. Moreover, we are also interested in asking whether an individual animal’s fear response to a given fearful event can be used to predict the animal’s responses to other fearful events. In order to address these questions, we set out to develop and calculate the fear resistance index by combining five individual HR parameters: (1) the rising speed (of the rising phase); (2) time duration of the maximum plateau; (3) HR of the maximum plateau; (4) CV of the maximum plateau; and (5) recovery speed (of the recovery phase). The fear resistance index, calculated from comprehensive evaluation index by using the above five factors based on the Shannon’s entropy method [43]–[45], is the quantitative measurement of summed responses to fearful events. The higher the fear resistance index, the lower the HR response magnitude of mice to fearful challenges (see methods for details).

**Figure 8:**
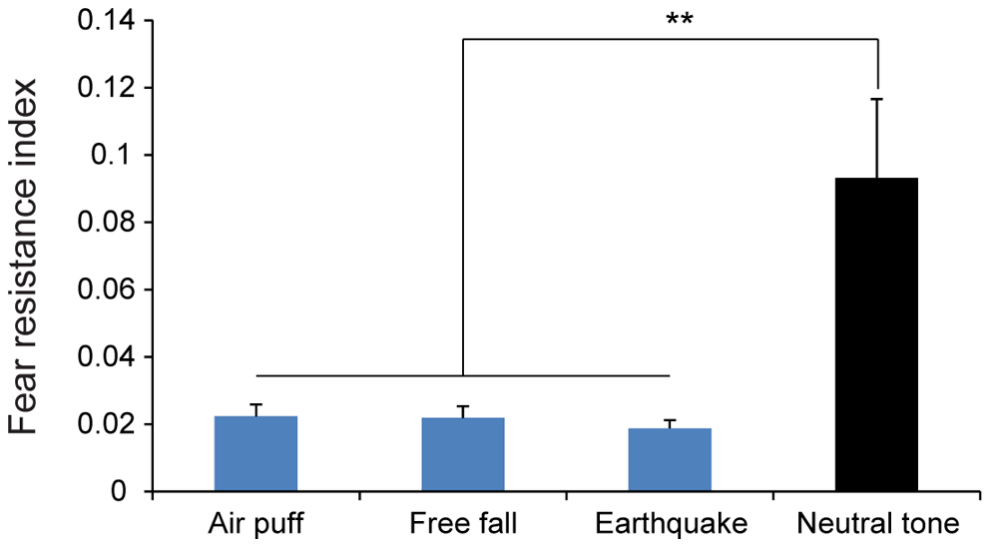
Comparison of fear resistance indexes between three types of fearful events and neutral tone. The fear resistance indexes during fearful events (n = 7) were significantly lower than those during neutral tone (n = 6). **P<0.01, one-way ANOVA and Tukey *post hoc* test. All data are plotted as mean ± SEM (error bars).

We first compared the fear resistance indexes during air puff, free fall and earthquake with those during neutral tone. As expected, the fear resistance indexes during fearful events were significantly lower than those during neutral tone (Figure 8, *F*_3, 23_ = 10.44, *P*<0.01), therefore providing evidence for the validity of such index.

**Figure 9:**
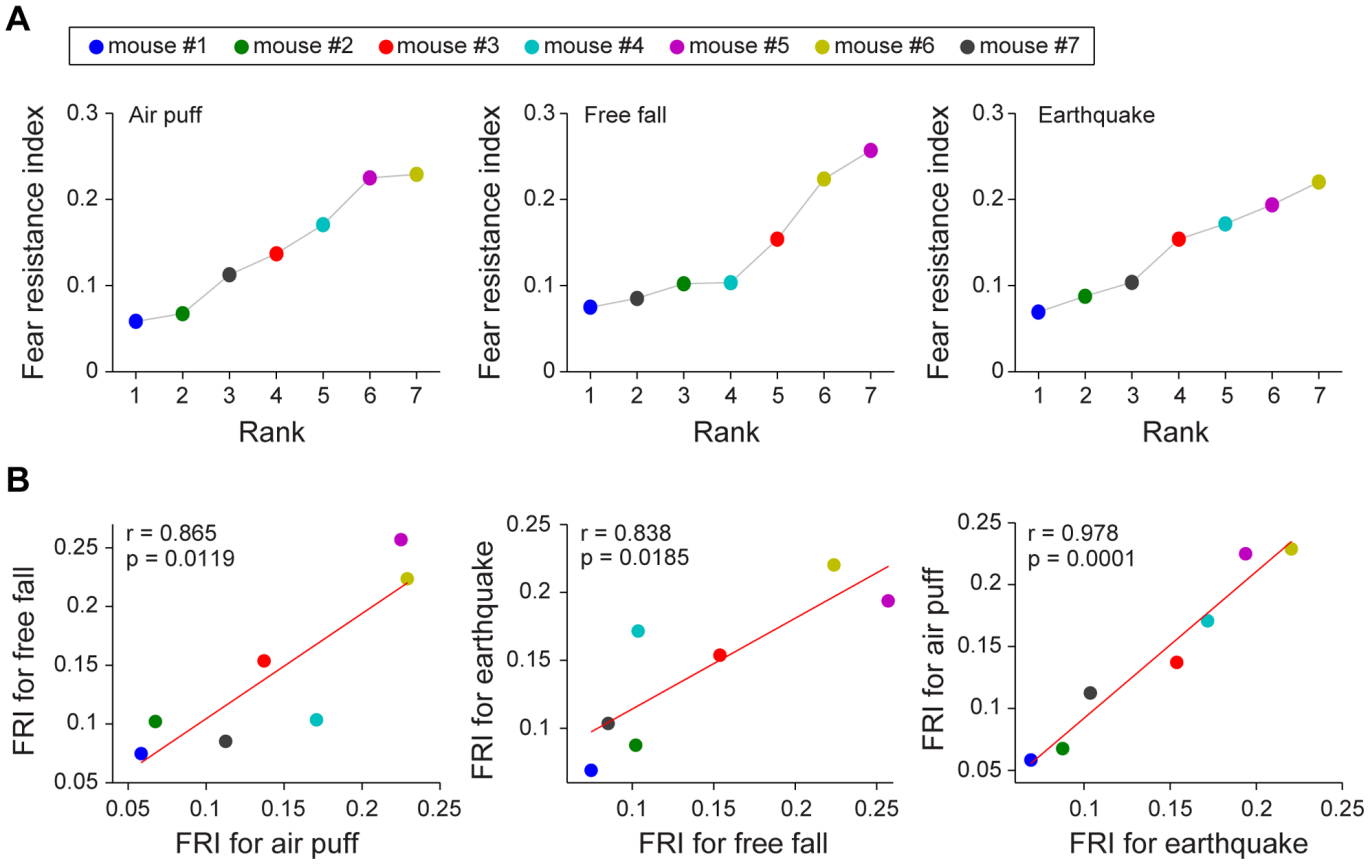
Consistency of cardiac responses to three different types of fearful stimuli in individual mice. (A) For each type of fearful stimuli (air puff, free fall, and earthquake, respectively), the fear resistance index (FRI) was developed and calculated from comprehensive evaluation index by combining five individual HR parameters. Individual mice were ranked in the order of the fear resistance index (air puff, the left panel; free fall, the middle panel; earthquake, the right panel). The fear resistance index of each mouse revealed the high consistency of cardiac responses to the three types of fearful stimuli in individual mice. (B) The regression analysis of fear resistance index (FRI) in seven mice between air puff and free fall (the left panel), between free fall and earthquake (the middle panel), and between earthquake and air puff (the right panel) was carried out respectively. Significant correlations were observed across all the three different types of fearful stimuli.

Next, we calculated the fear resistance indexes of the mice during air puff. Our analysis showed that Mouse #1, #2, and #7 were ranked as the top three (in order) for exhibiting the strongest HR responses to air puff, whereas Mouse #6 had the least response to sudden air puff and Mouse #5 the second least response (Figure 9A, the left panel). At the same time, Mouse #3 and #4 belonged to the middle of the group (Figure 9A, the left panel, resistance indexes as follows: mouse #1< #2< #7< #3< #4< #5< #6). Second, we calculated the summed responses to free fall. Interestingly, the fear resistance indexes obtained from free-fall drop experiments also showed the similar rank order with Mouse #1, #7, and #2 as the top three mice for exhibiting the strongest HR-related dynamics, and whereas mouse #4 and #3 in the middle and Mouse #6 and #5 had the least fearful responses (Figure 9A, the middle panel, mouse #1< #7< #2< #4< #3< #6< #5). Finally, we further calculated the summed responses to earthquake. We also found that the same group of mice exhibited the identical rank order of their fear resistance indexes for earthquake in comparison to those of air puff (Figure 9A, the right panel, mouse #1< #2< #7< #3< #4< #5< #6). These results suggest that fear responses exhibited by each individual mouse tended to be rather consistent when exposed to different fearful events, thereby indicating the possible epigenetic factors in defining and controlling fear responses in a given individual.

Further assessing this consistency of animals’ summed fearfulness across air puff, free fall, and earthquake, we found the summed fear responses from these seven mice during air puff and free fall exhibited significant correlation (r = 0.865, *P = *0.0119) (Figure 9B, the left panel). Similarly, the correlation between the fear resistance indexes for free fall responses and earthquake responses was also statistically significant (Figure 9B, the middle panel, r = 0.838, *P = *0.0185). In addition, the regression analysis also showed the tight correlation between fear resistance indexes to earthquake and air puff (Figure 9B, the right panel, r = 0.978, *P = *0.0001). These analyses again suggest the consistent cardiac responses of individual mice to different fearful stimuli.

## Discussion

Our above experiments provided a detailed characterization of changes in HR and HRV, and recovery speed in freely behaving mice subjected to three different types of fearful events. Our use of free fall paradigm has been prompted by our desire to understand how and why Bungee jump or the Tower of Terror ride at Disney World would create long-lasting memory in humans. While such experiences can be highly fearful, our recent study in VTA revealed that some dopamine neurons exhibited suppression-rebound excitation dynamics. This may explain why such events can be paradoxically rewarding [31]. The stimulation paradigms, such as free fall and earthquake, which are quite relevant to both humans and animals, have also expanded experimental protocols for the study of fear memory processing that was often traditionally relied on fear conditioning paradigms or air puff. Over the past several years, we have investigated *in vivo* neural activity in several mouse brain regions including the CA1, ACC and VTA during free fall, earthquake, and air puff [28]–[31]. We have shown that our novel behavioral protocols are quite useful to enrich our understanding how the hippocampus and other brain regions encode fear memories [29], [46]. Three major findings emerged from our present study: First, earthquake, free fall, and air puff all evoked significant changes in cardiac responses such as increased HR and decreased HRV. It is stated that HRV could be regarded as a reflection of moment-to-moment interplay between the two countervailing branches of the autonomic nervous system, namely an excitatory sympathetic component and an inhibitory parasympathetic component [47], [48]. Thus changes in HRV were associated with changes in the regulatory output via efferent nerve traffic of the autonomic nervous system to the sinoatrial node [38], [49]. In the literature, both increased HR and decreased HRV were typically observed in mice during acquisition as well as contextual and auditory cued retention phase of the aversive conditioning, a form of associative learning [37], [38], [42]. Such effect on HR during tone recall phase could be inhibited by the nonspecific beta-adrenergic antagonist sotalol, indicating a strong sympathetic activation [37]. Moreover, several lines of evidence suggested that reduced HRV but not HR could serve as a more sensitive marker of enhanced fear response [42], [47].

While we are not aware of any study conducted to measure HR and HRV during free fall and earthquake-like shake, several previous studies have reported the autonomic responses evoked by air puff and acoustic startle [32]–[36], [40], [41], [50]. It has been reported that alerting stimuli such as acoustic startle or air puff stimuli could elicit tachycardia in freely moving mice [26], [47], as well as in unrestrained rats [26]. However, some studies showed bradycardia in restrained rats when they were subjected to air puff, and such cardiac deceleration could be habituated over trials [33], [40], [41]. The cardiac responses to such alerting stimuli could reflect the dynamic changes in sympathetic and parasympathetic modulation under different behavioral states. In comparison, our finding in freely behaving mice showed a robust and consistent tachycardia to all the three fearful stimuli, but no bradycardia was observed. It is noteworthy to point out that upon the earthquake-like shake and drop, mice tended to move or escape. Thus, the traditional behavioral parameters such as the amount of freezing were not suitable for measuring fearful responses under these situations. The demonstration of changes in HR and HRV in mice should provide strong evidence for the notion that earthquake and free fall indeed represent fearful experiences to these rodents, just as these events to humans. This may, in turn, support the idea that these events can be and should be meaningful, not only ecologically, but also experimentally, to the study of fear memories beyond the traditional fear conditioning paradigms using context or cue associated with mild foot-shock.

Second, to further understand how cardiac physiology changes over repeated exposures to earthquake-like shake, free fall, or air puff to the back of the animals, we have divided cardiac dynamic responses into five distinct components, namely, the rising speed (of the rising phase), time duration of the maximum plateau, HR of the maximum plateau, CV of the maximum plateau, and recovery speed (of the recovery phase). Our systematic analyses have revealed that each of these five components contained useful information about the cardiac responses to these fearful events. These distinct components exhibited differential responses to habituation protocols. Specifically, the HR acceleration did not differ across trials. However, the HR and HRV of the maximum plateau phase showed significant differences upon the stimuli but remained relatively similar over all seven trials. Thus, these observations indicate that the slope of HR increase, increment of HR and decrement of HRV are important to reflect the cardiac response, but are not necessarily suitable to reflect the habituation effect. Furthermore, this may be different with the extinction of tachycardia triggered by conditioned stressor, during which the maximum HR decreased with repeated testing [51]. Interestingly, we found that time durations of the maximum plateau and the recovery speed were more sensitive to show such habituation effects. In addition, the highest baseline HR values and the largest baseline variation were noted during seven trials of free fall. The chamber needed to be raised vertically before each trial during free fall experiments, which could be the cause of the baseline fluctuation. Because the HR acceleration slowed down with higher HR [26], such fluctuation could lead to the variation in rising speed of HR (Figure 5A). Since the rising speed was one of the parameters for fear resistance index calculation, it should also result in the lower correlations in Figure 9 (left and middle panels).

Third, we employed a Shannon’s entropy method and developed a novel method to describe the summed responses using these five components of measured cardiac changes. We can provide each individual animal with its unique fear resistance index. This has enabled us not only to compare the individual response consistency in the same animal over the different types of fearful events, but also use the cardiac dynamic-based metrics for comparative studies across different animals. It is well known that there is remarkable inter-individual variability in human’s stress responses [52]. Complex interactions between genes and developmental experience could cause the variation in humans [52]. The present study has investigated the individual differences of cardiac responses to fearful stimuli in isogenic mice. Our result showed each individual mouse had consistent cardiac responses to different fearful stimuli. Furthermore, we noted significant differences among our individual wild-type mice (C57BL/6J). Although the inbred strains of mice, such as C57BL/6J mice are genetically homogeneous, the behavior in mice may result not only from genetics but also from environmental influences [53]. Several studies have showed the individual differences of behavior in the isogenic mice [54]–[57]. The prenatal period and post-natal period experiences, such as intrauterine position, nutrition in utero, maternal stress, and social status may exert certain influences [58], [59]. This suggests the possible epigenetic factors may contribute to such variability of cardiac responses to fearful stimuli in C57BL/6J mice. It is conceivable that the fear resistance index can be quite useful in future characterizations of neural responses in the mouse brain, especially in relationship with the processing of real-time fear memory traces associated with air puff, free fall and earthquake experiences [28]–[30].

In conclusion, we have shown the rapid increases in HR and great reduction in HRV as mice were subjected to fearful events such as earthquake, free fall, and air puff. By systematically characterizing five distinct components related to changes in HR and HRV, we show that the duration of the maximum plateau phase and HR recovery speed are quite sensitive to the habituation effects. Moreover, based on the measurement of the fear resistance index, we can compare the summed cardiac responses across different fearful events and across different animals. The isogenic mice (C57BL/6J) showed consistent differences on inter-individual fear response magnitudes. In addition, it is generally accepted that in affective disorders altered HR dynamics will increase the risk of cardiac mortality [49]. Therefore, the fear resistance index may open a door to measure personality traits or individual differences in stress-susceptibility in both wild-type mice and post-traumatic stress disorder (PTSD) models.

## Materials and Methods

### Ethics Statement

All animal work described in this study was carried out according to the National Institutes of Health Guide for Care and Use of Laboratory Animals, and was approved by the Institutional Animal Care and Use Committee of Georgia Regents University and Banna Biomedical Research Institute.

### Subjects

A total of seventeen adult male C57BL/6J mice were used for ECG recording. Mice were individually housed on a 12:12-h light-dark cycle (Lights on at 8:00 a.m.) and had access to food and water *ad libitum*. Data from thirteen mice in which the ECG signals were stably recorded were analyzed here.

### Animal Surgery and Electrocardiogram (ECG) Recording

Electrocardiogram (ECG) measurements were employed on freely behaving mice, similar to that previously described [42]. Briefly, mice were isolated from standard cages and singly housed in the round home cages (48 cm in diameter, 42 cm in height) one week before surgery and handled twice a day (5 min each session) to minimize the potential stress from the experimenter. On the day of surgery, the mouse was anesthetized with i.p. injection of Ketamine/Domitor (60/0.5 mg/kg). With the mouse placed supine on a heating pad, a single pair of insulated electrodes penetrated subcutaneously from the back of the neck to the chest in the lead II configuration [60]. The positive electrode was placed in the left abdomen below the diaphragm and anchored to the underlying peritoneal tissue, while the negative electrode was placed in the right upper chest and anchored to the pectoral muscle. The incisions were then carefully sutured. The mouse was awoken with an i.p. injection of Antisedan (2.5 mg/kg), and then returned to the home cage to allow for fully recovery (at least five days) before any of the behavioral tests were introduced.

The ECG electrodes were pre-attached to a miniature connector (Omnetics). The latter was then connected to Multichannel Acquisition Processor system (Plexon Inc., Dallas, TX) via ultra-fine 36 gauge wires (0.127 mm in diameter, Plexon Inc., Dallas, TX). A small helium-filled mylar balloon was tied to the middle of the wires to balance the weight. The wires were long enough (230 cm) so that the animal was able to move freely within the home cage and experiment chambers. The ECG signals (filtered at 0.7–300 Hz, digitized at 5 kHz) were monitored and recorded by using the Plexon Sort Client software, as well as the mice behaviors by using the Plexon CinePlex video recording and tracking system.

### Behavioral Paradigms

Three different types of fearful stimuli were designed and performed as previously described [28]. (1) A sudden air puff to the animal’s back (termed air puff, 10 psi, 0.5 s). The air tube (a vinyl tube, I.D. 1.5 mm) was bundled with ECG recording cable. Therefore, the air puff was consistently delivered to the animal’s back, when the animal was freely behaving in the home cage. (2) An abrupt drop of the animal (termed free fall, 13 cm high) inside a small elevator (a square chamber, 10×10×15 cm). (3) An unexpected earthquake-like shaking to the animal (termed earthquake, 3000 rpm, 0.5 s) inside a circular chamber (12.5 cm in diameter, 15 cm in height) fixed on a vortex mixer. To maintain the consistency of stimulus inputs and yet minimize possible prediction of upcoming stimuli, the fearful events were triggered using a computer. The air valve (ASCO Valve, Inc., SC 8202A206V), suspension system (Magnetic Sensor Systems, Series S-20-125), and vortex machine (Thermolyne Maxi Mix II Type 37600 Mixer) were precisely controlled in timing and intensity by the computer programs. The timestamps of the fearful events were recorded along with the ECG signal. To examine potential habituation effects, we delivered each type of fearful events to mice for seven times at randomized intervals varying between 2 and 5 min. The experiment sequence was as follows: earthquake, free fall, and air puff. The interval between sessions was 1 h. In order to minimize the effect of novel environment on HR [42], mice were habituated in both the free fall and earthquake chambers for 1 h per day, and three days in total. On the day of experiments, mice were further allowed to be habituated to the chambers for at least 15 min before free fall and earthquake stimuli started to deliver.

In a control experiment, a second group of mice were subjected to neutral tone (30 s, 85 dB, 5000 Hz) in the home cage seven times with 2–5 min randomized intervals.

### Analysis of Cardiac Response Dynamics

All the ECG analyses were conducted in Matlab by using custom-written codes. The R-wave maxima of ECG signals were obtained by peak detection algorithm to calculate the R-R intervals, and the R-R interval time series were converted into instant HR time series for analyses. Upon a fearful stimulus, the HR first increased dramatically (rising phase), and then entered into a maximum plateau phase during which HR remained high and HRV greatly reduced. At last, the HR gradually recovered to basal levels (recovery phase). Five HR model parameters (rising speed, plateau duration, HR of maximum plateau, CV of maximum plateau and recovery speed) were derived from these three phases.

For the maximum plateau detection, the point of maximal slope variation was chosen as the start of the maximum plateau. The momentary slope variation of the *n*th point (the 1st point was the onset of the stimulus) was calculated by the angle of two lines as follows: 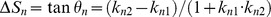, where *k_n1_* represents the slope of a line fitted to data points {*HR_n−4_*, *HR_n–3_*, *HR_n–2_*, *HR_n–1_*, *HR_n_*}, and *k_n2_* represents the slope of a line fitted to data points {*HR_n_*, *HR_n+1_*, *HR_n+2_*, *HR_n+3_*, *HR_n+4_*}. The linear least squares fitting method was used in the line fitting. Beginning with the start of the maximum plateau, the accumulative standard deviation was calculated with 1-point increment. The accumulative standard deviation as a function of time was derived. The accumulative standard deviation increased dramatically in the first a few points and then became steady. The sudden increase point after the steady state was defined as the end of the maximum plateau. The plateau duration was the time between the start and end of the maximum plateau. The HR of maximum plateau was the mean of instant HR during the maximum plateau phase. The CV of maximum plateau was based on the ratio of the standard deviation of R-R intervals *σ* to the mean of R-R intervals *μ* during the maximum plateau phase: 
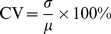
.

The rising phase was defined as the time between the onset of the stimulus and the start of the maximum plateau. The rising speed of instant HR was defined as the ratio of the elevation in HR to rising time. The elevation in HR was the difference between the instant HR of the first point of maximum plateau and the mean of instant HR during baseline period (ten seconds time period before the stimulus).

The recovery phase was specified as the time between the end of the maximum plateau and the time point when HR recovered to 1.1 times of the baseline. The mean of instant HR was calculated using a 5-s bin with a sliding window of 1-point after the maximum plateau. The recovery speed of instant HR was calculated by the ratio of the reduction in HR to recovery time.

We used the Poincaré plot analysis to visualize the dynamics of HRV. Each heartbeat interval, R-R interval_n_ was plotted on the X-axis against the subsequent heartbeat interval, R-R interval_n+1_ on the Y-axis.

Root Mean Square of the Successive Differences (RMSSD) is one of time-domain tools used to assess HRV, the successive differences being neighboring R-R intervals: 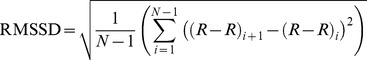, where (*R*-*R*)*_i_* represents the *i*th R-R intervals, *N* = number of R-R interval terms.

### Fear Resistance Index

To obtain the summed cardiac responses from a given mouse in response to a given fearful event, we calculated the weight of each of the five cardiac parameters by using the Shannon’s Entropy Weight Method. These five parameters are as follows: (1) the rising speed (of the rising phase) which characterizes the speed of the elevation of HR upon stimulation; (2) time duration of the maximum plateau; (3) HR of the maximum plateau; (4) CV of the maximum plateau; and (5) recovery speed (of the recovery phase).

Supposing there are *n* evaluation parameters and *m* sample data, the original data matrix 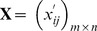 is formed. The main steps of using entropy coefficient method to determine the weights are as follows:

1. The standardization of original data. The raw data are standardized to eliminate anomalies with different measurement units, scales and positive-negative orientation. This process transforms different scales, units and orientation among various parameters into common measurable units to allow for comparisons of different parameters. The parameter playing a positive role: 
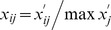
, where 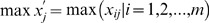. The parameter playing a negative role: 
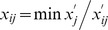
, where 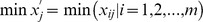. Then set 
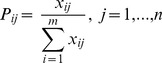
.
2. Computation of entropy. The entropy of the *j*th parameter was defined as follow: 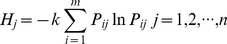, where *k* is the entropy constant and is equal to 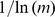.
3. Evaluation of parameter weight. The degree of diversification is calculated by 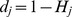. The weight of *j*th parameter is 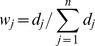, which indicated the degree of importance of parameter *j*.
4. Comprehensive evaluation value. The comprehensive evaluation value can be derived as 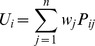.

Here, the comprehensive evaluation index, namely fear resistance index of different mice for three types of fearful stimuli, was calculated respectively. Take earthquake for example, seven sample data points from the first trial of seven mice and five parameters (rising speed, plateau duration, HR of the maximum plateau, CV of the maximum plateau and recovery speed) were used to form the evaluation matrix. The greater fear resistance index implied the less response of the mouse to earthquake. The higher rising speed (*j* = 1) indicated the stronger response to the stimulus; the longer the plateau duration (*j* = 2) showed the greater lasting effect of the stimulus; the higher HR of the maximum plateau (*j* = 3) implied the greater reaction to the stimulus; the larger value of the CV during the maximum plateau (*j* = 4) associated with the weaker response to stimulus; the slower recovery speed (*j* = 5) indicated the stronger effect of stimulus. We treated the rising speed, plateau duration and HR of the maximum plateau as reverse indexes; the CV of the maximum plateau and recovery speed were treated as positive indexes. The cardiac responses from each mouse can be ranked according to the fear resistance index resulting from entropy method. As a result, three ranks based on fear resistance index can be derived from each mouse in terms of their response to air puff, free fall and earthquake stimuli, respectively. The correlation coefficient analysis was conducted in Matlab using “corrcoef” function.

For comparing fear resistance indexes between three types of fearful events and neutral tone, 27 sample data points in total (including 21 sample data points from the first trial of each fearful stimulus of 7 mice, and 6 sample data points from the first trial of neutral tone) were used to form the evaluation matrix to get the comprehensive evaluation value.

### Statistical Analysis

For the comparisons of multiple means, one-way ANOVA, one-way repeated measures ANOVA and Tukey *post hoc* tests were conducted to assess the difference of means. Dunnett’s multiple comparisons test was also performed to compare the means at individual time points to the control (first trial). Data are presented as mean ± SEM. Differences were considered significant if *P* values were <0.05.

## References

[1] LeDoux JE (1994) Emotion, memory and the brain. Scientific American 270: 50–57. 10.1038/scientificamerican0694-50 8023118

[2] Koch M (1999) The neurobiology of startle. Prog Neurobiol 59: 107–128. 10463792 10.1016/s0301-0082(98)00098-7

[3] LaBar KS, Cabeza R (2006) Cognitive neuroscience of emotional memory. Nat Rev Neurosci 7: 54–64. 16371950 10.1038/nrn1825

[4] Bradley MM, Greenwald MK, Petry MC, Lang PJ (1992) Remembering pictures: pleasure and arousal in memory. J Exp Psychol Learn Mem Cogn 18: 379–390. 1532823 10.1037//0278-7393.18.2.379

[5] Cahill L, McGaugh JL (1998) Mechanisms of emotional arousal and lasting declarative memory. Trends Neurosci 21: 294–299. 9683321 10.1016/s0166-2236(97)01214-9

[6] Adolphs R, Tranel D, Denburg N (2000) Impaired emotional declarative memory following unilateral amygdala damage. Learn Mem 7: 180–186. 10837507 10.1101/lm.7.3.180 PMC311327

[7] Phelps EA (2004) Human emotion and memory: interactions of the amygdala and hippocampal complex. Curr Opin Neurobiol 14: 198–202. 15082325 10.1016/j.conb.2004.03.015

[8] Landis C, Hunt WA (1939) The startle pattern: Farrar and Rinehart, New York.

[9] Blanchard DC, Blanchard RJ (1972) Innate and conditioned reactions to threat in rats with amygdaloid lesions. J Comp Physiol Psychol 81: 281–290. 5084445 10.1037/h0033521

[10] Fanselow MS (1984) What is conditioned fear. Trends in Neurosciences 7: 460–462.

[11] Davis M (1984) The mammalian startle response. In: Eaton RC, editor. Neural Mechanisms of Startle Behavior. New York: Plenum Press. 287–351.

[12] Berger TW, Thompson RF (1978) Identification of pyramidal cells as the critical elements in hippocampal neuronal plasticity during learning. Proc Natl Acad Sci U S A 75: 1572–1576. 274742 10.1073/pnas.75.3.1572 PMC411516

[13] Kim JJ, Fanselow MS (1992) Modality-specific retrograde amnesia of fear. Science 256: 675–677. 1585183 10.1126/science.1585183

[14] Clark RE, Squire LR (1998) Classical conditioning and brain systems: the role of awareness. Science 280: 77–81. 9525860 10.1126/science.280.5360.77

[15] Maren S (2001) Neurobiology of Pavlovian fear conditioning. Annu Rev Neurosci 24: 897–931. 11520922 10.1146/annurev.neuro.24.1.897

[16] Weible AP, Weiss C, Disterhoft JF (2003) Activity profiles of single neurons in caudal anterior cingulate cortex during trace eyeblink conditioning in the rabbit. J Neurophysiol 90: 599–612. 12750412 10.1152/jn.01097.2002

[17] Kim JJ, Jung MW (2006) Neural circuits and mechanisms involved in Pavlovian fear conditioning: a critical review. Neurosci Biobehav Rev 30: 188–202. 16120461 10.1016/j.neubiorev.2005.06.005 PMC4342048

[18] Lang PJ (1995) The emotion probe. Studies of motivation and attention. Am Psychol 50: 372–385. 7762889 10.1037//0003-066x.50.5.372

[19] Bradley MM, Cuthbert BN, Lang PJ (1999) Affect and the startle reflex. Startle modification: Implications for neuroscience, cognitive science, and clinical science: 157–183.

[20] Brown JS, Kalish HI, Farber IE (1951) Conditioned fear as revealed by magnitude of startle response to an auditory stimulus. J Exp Psychol 41: 317–328. 14861383 10.1037/h0060166

[21] Chi C (1965) The effect of amobarbital sodium on conditioned fear as measured by the potentiated startle response in rats. Psychopharmacologia 7: 115–122. 5830969 10.1007/BF00403634

[22] Miserendino MJD, Sananes CB, Melia KR, Davis M (1990) Blocking of acquisition but not expression of conditioned fear-potentiated startle by NMDA antagonists in the amygdala. Nature 345: 716–718. 1972778 10.1038/345716a0

[23] Davis M, Falls WA, Campeau S, Kim M (1993) Fear-potentiated startle: a neural and pharmacological analysis. Behav Brain Res 58: 175–198. 8136044 10.1016/0166-4328(93)90102-v

[24] Ruiz-Padial E, Sollers JJ 3rd, Vila J, Thayer JF (2003) The rhythm of the heart in the blink of an eye: emotion-modulated startle magnitude covaries with heart rate variability. Psychophysiology 40: 306–313. 12820871 10.1111/1469-8986.00032

[25] Davis M, Gendelman DS, Tischler MD, Gendelman PM (1982) A primary acoustic startle circuit: lesion and stimulation studies. J Neurosci 2: 791–805. 7086484 10.1523/JNEUROSCI.02-06-00791.1982 PMC6564345

[26] Tovote P, Meyer M, Pilz PK, Ronnenberg A, Ogren SO, et al (2005) Dissociation of temporal dynamics of heart rate and blood pressure responses elicited by conditioned fear but not acoustic startle. Behav Neurosci 119: 55–65. 15727512 10.1037/0735-7044.119.1.55

[27] Vogel EH, Wagner AR (2005) Stimulus specificity in the habituation of the startle response in the rat. Physiol Behav 86: 516–525. 16199066 10.1016/j.physbeh.2005.08.042

[28] Lin L, Osan R, Shoham S, Jin W, Zuo W, et al (2005) Identification of network-level coding units for real-time representation of episodic experiences in the hippocampus. Proc Natl Acad Sci U S A 102: 6125–6130. 15833817 10.1073/pnas.0408233102 PMC1087910

[29] Osan R, Chen G, Feng R, Tsien JZ (2011) Differential consolidation and pattern reverberations within episodic cell assemblies in the mouse hippocampus. PLoS One 6: e16507. 21347227 10.1371/journal.pone.0016507 PMC3039647

[30] Xie K, Kuang H, Tsien JZ (2013) Mild blast events alter anxiety, memory, and neural activity patterns in the anterior cingulate cortex. PLoS One 8: e64907. 23741416 10.1371/journal.pone.0064907 PMC3669016

[31] Wang DV, Tsien JZ (2011) Convergent processing of both positive and negative motivational signals by the VTA dopamine neuronal populations. Plos One 6: e17047. 21347237 10.1371/journal.pone.0017047 PMC3039659

[32] Rettig R, Geyer MA, Printz MP (1986) Cardiovascular concomitants of tactile and acoustic startle responses in spontaneously hypertensive and normotensive rats. Physiol Behav 36: 1123–1128. 3725916 10.1016/0031-9384(86)90489-0

[33] Casto R, Nguyen T, Printz MP (1989) Characterization of cardiovascular and behavioral responses to alerting stimuli in rats. Am J Physiol 256: R1121–1126. 2719154 10.1152/ajpregu.1989.256.5.R1121

[34] Young BJ, Leaton RN (1994) Fear potentiation of acoustic startle stimulus-evoked heart rate changes in rats. Behav Neurosci 108: 1065–1079. 7893399 10.1037//0735-7044.108.6.1065

[35] Baudrie V, Tulen JH, Blanc J, Elghozi JL (1997) Autonomic components of the cardiovascular responses to an acoustic startle stimulus in rats. J Auton Pharmacol 17: 303–309. 9427109 10.1046/j.1365-2680.1997.00465.x

[36] Baudrie V, Laude D, Chaouloff F, Elghozi JL (2001) Genetic influences on cardiovascular responses to an acoustic startle stimulus in rats. Clin Exp Pharmacol Physiol 28: 1096–1099. 11903324 10.1046/j.1440-1681.2001.03593.x

[37] Stiedl O, Spiess J (1997) Effect of tone-dependent fear conditioning on heart rate and behavior of C57BL/6N mice. Behav Neurosci 111: 703–711. 9267648 10.1037//0735-7044.111.4.703

[38] Stiedl O, Tovote P, Ogren SO, Meyer M (2004) Behavioral and autonomic dynamics during contextual fear conditioning in mice. Auton Neurosci 115: 15–27. 15507402 10.1016/j.autneu.2004.07.006

[39] Zhang WN, Murphy CA, Feldon J (2004) Behavioural and cardiovascular responses during latent inhibition of conditioned fear: measurement by telemetry and conditioned freezing. Behav Brain Res 154: 199–209. 15302126 10.1016/j.bbr.2004.02.016

[40] Taylor BK, Printz MP (1996) Habituation of airpuff-elicited cardiovascular responses in the spontaneously hypertensive rat. Physiol Behav 60: 919–925. 8873270 10.1016/0031-9384(96)00154-0

[41] Palmer AA, Printz MP (1999) Strain differences in Fos expression following airpuff startle in Spontaneously Hypertensive and Wistar Kyoto rats. Neuroscience 89: 965–978. 10199628 10.1016/s0306-4522(98)00333-9

[42] Liu J, Wei W, Kuang H, Zhao F, Tsien JZ (2013) Changes in heart rate variability are associated with expression of short-term and long-term contextual and cued fear memories. PLoS One 8: e63590. 23667644 10.1371/journal.pone.0063590 PMC3646801

[43] Shannon CE (1948) A mathematical theory of communication. Bell Syst Tech J: 379–423.

[44] Fang FSC, Rajasekera JR, Tsao HSJ (1997) Entropy optimization and mathematical programming: Kluwer Academic Publishers.

[45] Islam S, Roy TK (2006) A new fuzzy multi-objective programming: Entropy based geometric programming and its application of transportation problems. European Journal of Operational Research 173: 387–404.

[46] Tsien JZ, Li M, Osan R, Chen G, Lin L, et al (2013) On initial brain activity mapping of episodic and semantic memory code in the hippocampus. Neurobiol Learn Mem 105: 200–210. 23838072 10.1016/j.nlm.2013.06.019 PMC3769419

[47] Gaburro S, Stiedl O, Giusti P, Sartori SB, Landgraf R, et al (2011) A mouse model of high trait anxiety shows reduced heart rate variability that can be reversed by anxiolytic drug treatment. Int J Neuropsychopharmacol 14: 1341–1355. 21320392 10.1017/S1461145711000058 PMC3198175

[48] Appelhans BM, Luecken LJ (2006) Heart rate variability as an index of regulated emotional responding. Review of general psychology 10: 229.

[49] Stiedl O, Jansen RF, Pieneman AW, Ogren SO, Meyer M (2009) Assessing aversive emotional states through the heart in mice: implications for cardiovascular dysregulation in affective disorders. Neurosci Biobehav Rev 33: 181–190. 18824021 10.1016/j.neubiorev.2008.08.015

[50] Eder DN, Elam M, Wallin BG (2009) Sympathetic nerve and cardiovascular responses to auditory startle and prepulse inhibition. Int J Psychophysiol 71: 149–155. 18824200 10.1016/j.ijpsycho.2008.09.001

[51] Stiedl O, Radulovic J, Lohmann R, Birkenfeld K, Palve M, et al (1999) Strain and substrain differences in context- and tone-dependent fear conditioning of inbred mice. Behav Brain Res 104: 1–12. 11125727 10.1016/s0166-4328(99)00047-9

[52] Ellis BJ, Jackson JJ, Boyce WT (2006) The stress response systems: Universality and adaptive individual differences. Developmental Review 26: 175–212.

[53] Francis DD, Szegda K, Campbell G, Martin WD, Insel TR (2003) Epigenetic sources of behavioral differences in mice. Nature Neuroscience 6: 445–446. 12665797 10.1038/nn1038

[54] Abreu-Villaça Y, Queiroz-Gomes FdE, Dal Monte AP, Filgueiras CC, Manhães AC (2006) Individual differences in novelty-seeking behavior but not in anxiety response to a new environment can predict nicotine consumption in adolescent C57BL/6 mice. Behavioural brain research 167: 175–182. 16214235 10.1016/j.bbr.2005.09.003

[55] Jakovcevski M, Schachner M, Morellini F (2008) Individual variability in the stress response of C57BL/6J male mice correlates with trait anxiety. Genes, Brain and Behavior 7: 235–243. 10.1111/j.1601-183X.2007.00345.x 17680803

[56] Crabbe JC, Wahlsten D, Dudek BC (1999) Genetics of mouse behavior: Interactions with laboratory environment. Science 284: 1670–1672. 10356397 10.1126/science.284.5420.1670

[57] Wahlsten D, Metten P, Phillips TJ, Boehm SL, Burkhart-Kasch S, et al (2003) Different data from different labs: Lessons from studies of gene-environment interaction. Journal of Neurobiology 54: 283–311. 12486710 10.1002/neu.10173

[58] Holmes A, le Guisquet AM, Vogel E, Millstein RA, Leman S, et al (2005) Early life genetic, epigenetic and environmental factors shaping emotionality in rodents. Neuroscience and Biobehavioral Reviews 29: 1335–1346. 16095695 10.1016/j.neubiorev.2005.04.012

[59] Lathe R (2004) The individuality of mice. Genes Brain and Behavior 3: 317–327. 10.1111/j.1601-183X.2004.00083.x 15544575

[60] McCauley MD, Wehrens XH (2010) Ambulatory ECG recording in mice. J Vis Exp. doi: 10.3791/1739. 10.3791/1739 PMC3152865 20517202

